# Efficacy of an internet-based exposure treatment for flying phobia (*NO-FEAR Airlines*) with and without therapist guidance: a randomized controlled trial

**DOI:** 10.1186/s12888-019-2060-4

**Published:** 2019-03-06

**Authors:** Daniel Campos, Juana Bretón-López, Cristina Botella, Adriana Mira, Diana Castilla, Sonia Mor, Rosa Baños, Soledad Quero

**Affiliations:** 1 Universitat Jaume I, Av. Vicente Sos Baynat s/n, 12006 Castellón, Spain; 2 CIBER de Fisiopatología de la Obesidad y Nutrición (CIBEROBN), Madrid, Spain; 3 Universitat de València, Valencia, Spain; 4 Department of Psychology and Sociology, Area of Psychobiology, University of Zaragoza, IIS Aragón, Teruel, Spain

**Keywords:** Internet-based exposure, Therapist guidance, Randomized controlled trial, Flying phobia, Self-help

## Abstract

**Background:**

Internet-based treatments appear to be a promising way to enhance the in vivo exposure approach, specifically in terms of acceptability and access to treatment. However, the literature on specific phobias is scarce, and, as far as we know, there are no studies on Flying Phobia (FP). This study aims to investigate the effectiveness of an Internet-based exposure treatment for FP (*NO-FEAR Airlines)* that includes exposure scenarios composed of images and sounds, versus a waiting-list control group. A secondary aim is to explore two ways of delivering *NO-FEAR Airlines*, with and without therapist guidance.

**Methods:**

A randomized controlled trial (RCT) was conducted in which 69 participants were allocated to: 1) *NO-FEAR Airlines* totally self-applied, 2) *NO-FEAR Airlines* with therapist guidance, 3) a waiting-list control group. Primary outcome measures were the *Fear of Flying Questionnaire-II* and the *Fear of Flying Scale*. Secondary outcomes included the *Fear and Avoidance Scales*, *Clinician Severity Scale,* and *Patient’s Improvement scale*. *Behavioral outcomes* (post-treatment flights and safety behaviors) were also included. Mixed-model analyses with no ad hoc imputations were conducted for primary and secondary outcome measures.

**Results:**

*NO-FEAR Airlines* (with and without therapist guidance) was significantly effective, compared to the waiting list control group, on all primary and secondary outcomes (all *p*s < .05), and no significant differences were found between the two ways of delivering the intervention. Significant improvements on diagnostic status and reliable change indexes were also found in both treatment groups at post-treatment. Regarding behavioral outcomes, significant differences in safety behaviors were found at post-treatment, compared to the waiting list. Treatment gains were maintained at 3- and 12-month follow-ups.

**Conclusion:**

FP can be treated effectively via the Internet. *NO-FEAR Airlines* helps to enhance the exposure technique and provide access to evidence-based psychological treatment to more people in need. These data are congruent with previous studies highlighting the usefulness of computer-assisted exposure programs for FP, and they contribute to the literature on Internet-based interventions. To the best of our knowledge, this is the first RCT to investigate the effectiveness of an Internet-based treatment for FP and explore two ways of delivering the intervention (with and without therapist guidance).

**Trial registration:**

Clinicaltrials.gov: NCT02298478 (https://clinicaltrials.gov/ct2/show/NCT02298478). Trial registration date 3 November 2014.

**Electronic supplementary material:**

The online version of this article (10.1186/s12888-019-2060-4) contains supplementary material, which is available to authorized users.

## Background

Flying phobia (FP) is a disabling disorder classified as a specific situational phobia [1], although authors also highlight its heterogeneous nature because FP symptoms can be influenced by many other fears [2–4]. Up to 7% of the population experience acute interference in daily and social life functioning due to FP [5]. Furthermore, around a quarter of the population (25%) suffer from anxiety when taking a flight, approximately 20% depend on alcohol or anxiolytics to fly, and about 10% avoid flying due to the intensity of their fear [6].

Research to date has pointed out that the most effective treatment approach for specific phobias (including FP) is in vivo exposure, recommending it as the treatment of choice [4, 7]. Specifically for FP, evidence indicates that more than 90% of participants whose treatment included in vivo exposure continued to fly at one- to four-year follow-up [8]. Despite this evidence, the in vivo exposure technique is linked to several limitations in its implementation, such as low acceptance among therapists and patients and difficulties in accessing the treatment. Regarding acceptance, some authors have considered in vivo exposure to be a cruel cure and ethically inappropriate [9, 10]. Around 25% of patients reject starting the treatment when they are informed about the procedure, or they drop-out during treatment because they consider it too aversive [11]. In terms of accessibility, only 7.8% of people suffering from phobias seek help [12], and very few of them (8%) receive a specific treatment for their problem [13]. In addition, in vivo exposure involves lack of confidentiality and high associated costs when conducted outside the therapist’s office [14]. Two issues that are particularly important in FP treatment are the economic cost of in vivo exposure and the additional difficulty of applying the exposure technique in an appropriate way (controlling important variables such as the duration of the exposure or the number of sessions) - due to the limited access to the feared stimulus (i.e., airport or airplane) [15].

Therefore, there is a demand for better types of exposure therapy. Specifically for FP, there is a need to improve the adherence, acceptance, and accessibility of the exposure therapy. Information and Communication Technologies (ICTs) can be useful for overcoming these issues, for example, through computerized treatments such as virtual reality exposure therapy (VRET) and computer-assisted exposure programs. The efficacy of VRET has been shown in several meta-analyses and systematic reviews for the treatment of anxiety disorders [16–19], including FP [6, 14, 20]. However, some authors suggest that less sophisticated and cheaper devices might be sufficient to produce satisfactory outcomes in FP [21]. Thus, Tortella-Feliu et al. [22] showed that a computer-assisted exposure program was as effective as VRET in FP treatment. Moreover, no significant differences were found between two ways of delivering this computer-assisted exposure treatment (with therapist assistance throughout the exposure vs. self-administered in the lab). According to these authors, the data also suggest that therapist involvement might be minimized in FP treatment using computer-assisted exposure programs.

An additional approach to using ICTs is to deliver psychological treatments over the Internet. In the past decade, the Internet has been established as a useful and effective tool to treat several psychological disorders [23–25]. Particularly for anxiety disorders, Internet-based treatments are highly effective and show comparable clinical outcomes to face-to-face treatment and large effect sizes compared to control groups (waiting list or placebo treatment) [26–28]. Moreover, authors especially recommend the use of self-applied interventions via the Internet for anxiety disorders because of their numerous advantages, including greater accessibility, versatility, safety, anonymity, acceptability, convenience, and cost-effectiveness [29–32].

Despite these findings and recommendations, research on Internet-based treatments for specific phobias is still scare. To date, the literature reviewed shows two randomized controlled trials (RCT), one on spider phobia [33] and one on snake phobia [34]. Similarly, Botella et al. [35] showed preliminary data from a series of cases about a self-applied telepsychology program using an intranet to treat small animal phobia (spiders, cockroaches, and mice). Moreover, other studies have pointed out the efficacy of Internet-based treatments for several disorders, including specific phobia. One example is the study by Kok, van Straten, Beekman and Cuijpers [36] who examined the efficacy of an Internet-based exposure intervention with weekly support for outpatients waiting for face-to-face psychotherapy for several phobias. In addition, several studies have tested the FearFighter™ program [37] for the treatment of panic and phobia disorders [38–40], which is used in the mental health services in England [41]. Finally, from a transdiagnostic perspective, Schöder, Jelinek and Moritz [42] conducted a randomized controlled trial of an Internet intervention for individuals with panic and phobias.

Regarding studies designed for specific phobia treatments, Andersson et al. [33, 34] found large within-group effect sizes for self-administered Internet treatments guided by the therapist from a distance, although in both studies the one-session exposure treatment (OST) was more effective than the self-administered Internet interventions. Nevertheless, as the authors noted, it is important to take into account that the Internet treatments used in both studies consisted of self-administered exposure, rather than a treatment delivered through a computer [34]. These treatments were mainly provided in the form of downloadable pdf files and a video sent to participants illustrating the exposure principles. Internet-based treatments usually include guidelines for exposure to the feared situations (i.e., downloadable pdf files), but without providing significant exposure stimuli (i.e., self-administered exposure scenarios through the computer). As some authors suggest, this may be especially relevant in treating specific phobias and other anxiety disorders [43, 44]. In line with the recommendations made by Botella et al. [43], we suggest that the combination of new technologies (i.e., multimedia exposure scenarios) and self-help procedures could be a useful clinical tool for the treatment of other psychological disorders, such as FP.

An important research issue in psychological treatments delivered via the Internet is the impact of guidance. Meta-analyses and systematic reviews have shown the beneficial feature of providing guidance throughout the intervention because it leads to better adherence and outcomes [45–48]. Although the literature suggests that the qualifications of those providing guidance (technicians vs. clinicians) might be of minor importance [46], some evidence highlights the superiority of guided interventions over unguided interventions [48]. Nevertheless, authors have recently shown that the magnitude of these differences is smaller than what was suggested in previous meta-analyses [46]. In addition, studies have pointed out that self-guided interventions are useful alternatives with similar outcomes that might work using automated reinforcement and no human support [49–52]. Despite these findings, there is no research on this issue in specific phobias, revealing the need for further research on this topic, particularly in FP.

In sum, there is a growing body of evidence about the effectiveness of Internet-based treatments to treat psychological disorders. However, the literature on specific phobias is scarce in this regard, and few studies have focused on the usefulness of the Internet in delivering systematic exposure through the computer. To the best of our knowledge, no published RCT has tested the efficacy of an Internet-based exposure treatment for FP. Therefore, the aim of this study is to investigate the effectiveness of an Internet-based exposure treatment for FP (*NO-FEAR Airlines)* that includes exposure scenarios composed of images and real sounds, versus a waiting list control group, in a randomized controlled trial (RCT). A secondary aim is to explore two ways of delivering *NO-FEAR Airlines*, with and without therapist guidance.

## Methods

### Study design

**Fig. 1:**
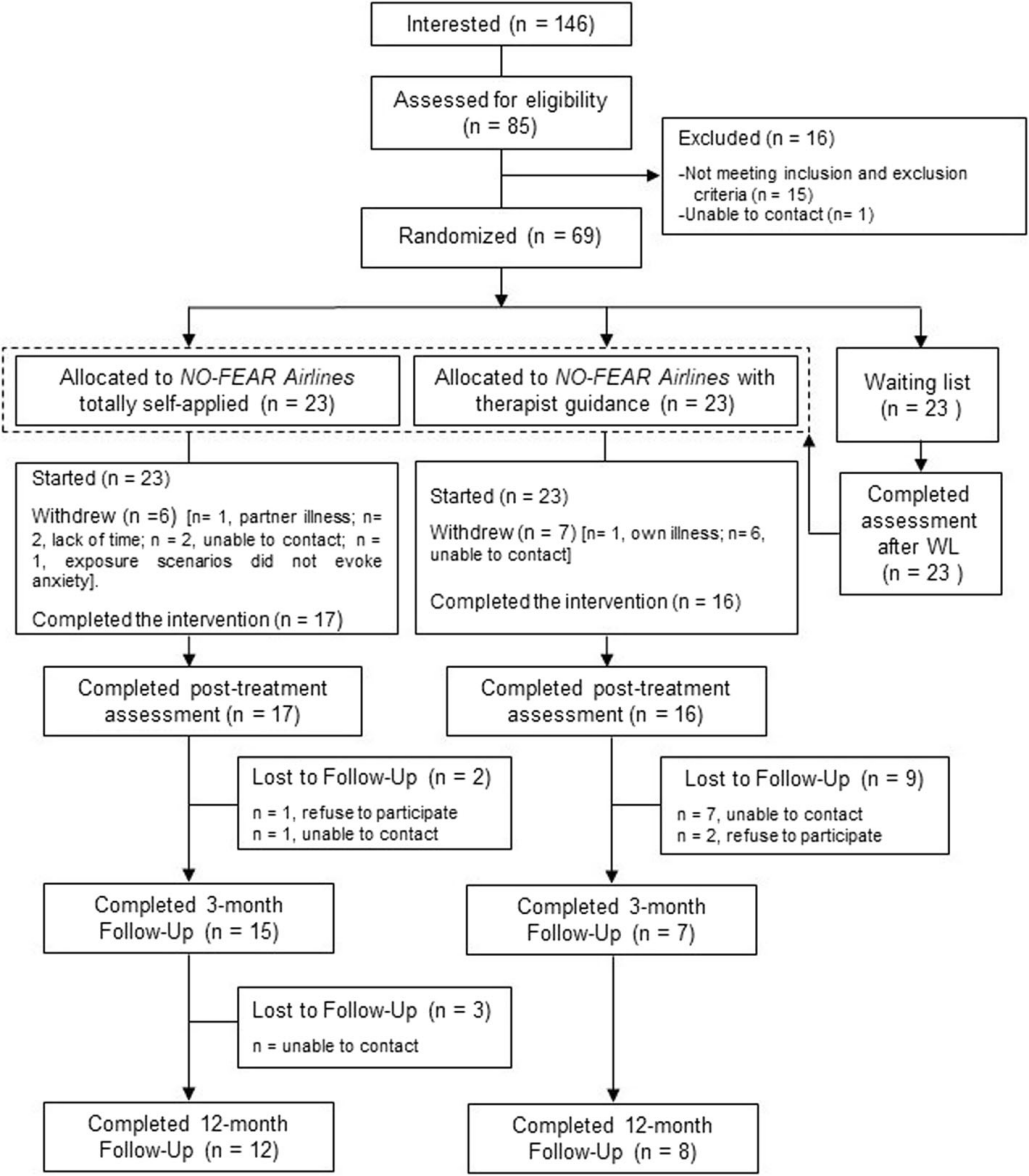
Flowchart

This study was a randomized controlled trial (RCT), in which participants were randomly allocated to three groups: 1) Internet-based exposure treatment for FP without therapist guidance (*NO-FEAR Airlines* totally self-applied, NFA); 2) Internet-based exposure treatment for FP with therapist guidance (*NO-FEAR Airlines* with therapist guidance, NFA + TG); and 3) a waiting list (WL) control group. For ethical reasons, participants in the WL group were randomly assigned to one of the two treatment conditions after spending time on the waiting list (6 weeks), thus leaving no control group for the follow-up measurements. Therefore, 3- and 12-month follow-up assessments were carried out for the two intervention groups (NFA and NFA + TG). The trial was registered at ClinicalTrials.gov (NCT02298478) on November 3, 2014. This trial received approval from the Ethics Committee of Universitat Jaume I (Castellón, Spain) (20 December 2014) and was conducted in compliance with the study protocol, following the CONSORT statement (Consolidated Standards Of Reporting Trials, http://www.consort-statement.org), the CONSORT-EHEALTH guidelines [53], the APA guidelines for the practice of telepsychology [54], the Declaration of Helsinki, and good clinical practice. Details of the study protocol have been reported elsewhere [55]. Changes in the original protocol were made related to the procedure for handling missing data. Intent-to-treat (ITT) mixed-model analyses without any ad hoc imputations were conducted, rather than using analysis of variance (ANOVA) with multiple imputations (MI), based on the authors’ recommendation and due to the large amount of missing data at follow-up [56, 57]. Figure 1 shows the flow diagram.

### Participants, recruitment and randomization

The study was advertised online via professional websites (i.e., LinkedIn), non-professional social-networks (i.e., Facebook and twitter), and announcements placed in local universities and in the local media (newspapers and radios). People who were interested in participating in the study registered on the website [58] and signed the informed consent form. The clinical team contacted participants by telephone or through the *skype* platform in the case of international calls, in order to screen accessibility criteria and explain the terms of the research. The inclusion criteria were: a) being at least 18 years old; b) meeting DSM-5 criteria for specific, situational phobia (FP) [1]; c) having adequate knowledge to understand and read Spanish; d) having access to the Internet; e) and ability to use a computer. The exclusion criteria were: a) receiving psychological treatment for FP; b) diagnosis of a severe mental disorder (abuse or dependence on alcohol or other substances, psychotic disorder, dementia, or bipolar disorder); c) presence of depressive symptomatology, suicidal ideation or plan; d) presence of heart disease; e) and pregnant women (from the fourth month). Participants with comorbid and related disorders (i.e., panic disorder, agoraphobia, claustrophobia, or acrophobia) were included when FP was the primary diagnosis. Receiving pharmacological treatment was not an exclusion criterion, but any increase and/or change in the medication during the study period implied the participant’s exclusion from subsequent analyses. A decrease in pharmacological treatment was accepted.

Participants who meet the criteria were administered a baseline telephone assessment that included the diagnostic interview. After that, they were randomly assigned to one of the three experimental groups (*n* = 69). A computer-generated randomization list was created using the Epidat 4.0 program [59], by an independent researcher who was unaware of the characteristics of the study and had no clinical involvement in the trial or access to the study data. The allocation scheme was communicated to clinicians via a phone call. In the same way, researchers contacted participants to explain the condition to which they had been allocated, and access to the program was provided if required. Thus, researchers and participants were blind to the experimental condition during the assessment at baseline, and patients agreed to participate before knowing the random allocation. However, they could not be blind to the treatment conditions for practical reasons. Participants were free at any time to withdraw from the study without giving any explanation. Access and participation in the study did not involve payment in any case.

### Intervention

**Fig. 2:**
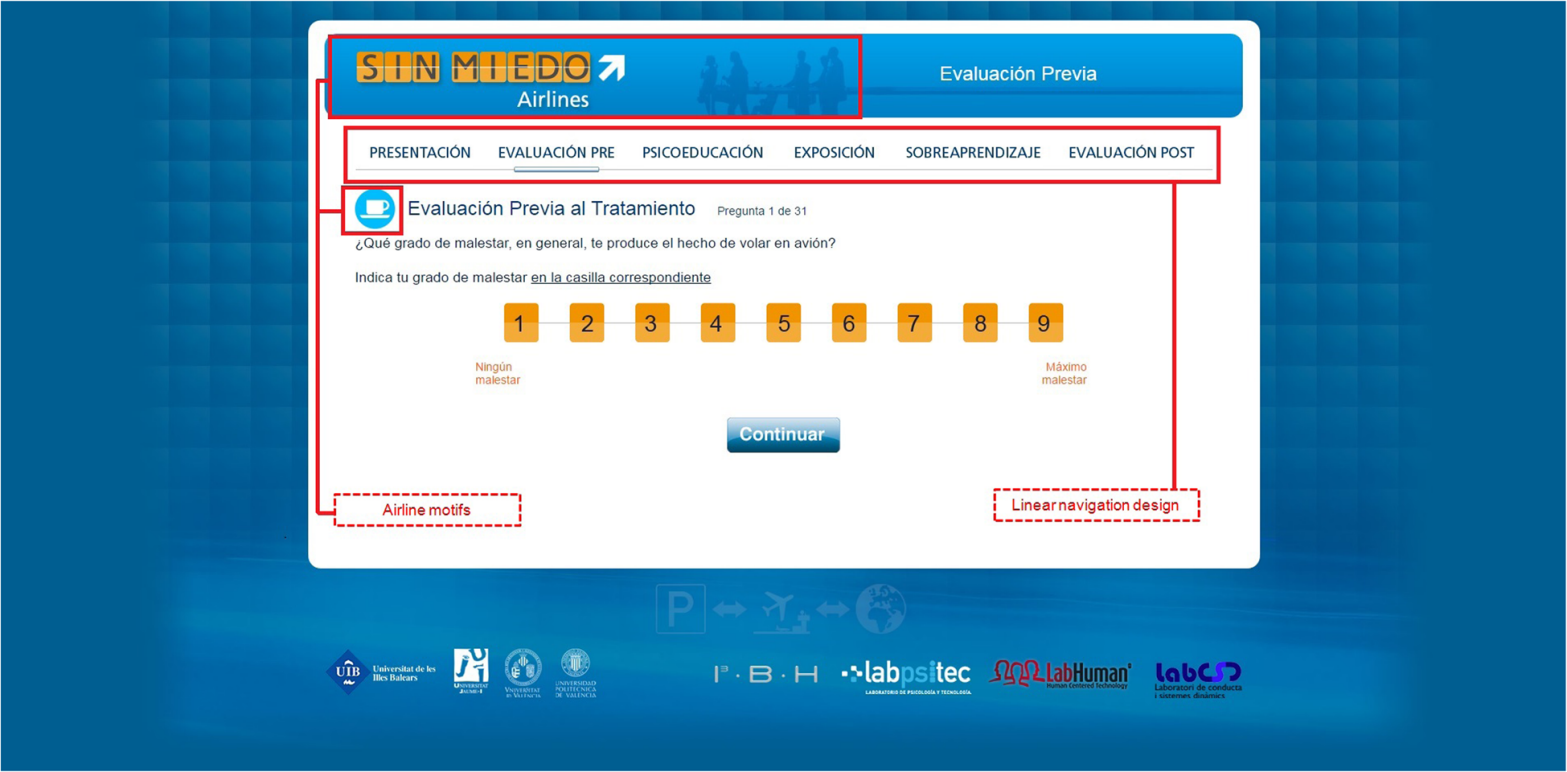
NO-FEAR Airlines “screenshot”: Linear navigation design and Airline motif examples

*NO-FEAR Airlines* is a computer-aided exposure treatment for FP that can be completely self-applied via the Internet [55, 60] (see Additional file 1). This Internet-based intervention allows people who are afraid of flying to be exposed to images and real sounds related to their phobic fears on a standard personal computer. The graphical user interface was designed according to visual flying metaphors (i.e., Airline motifs) and with linear navigation, in order to optimize the treatment structure and make the treatment easier and more attractive to the users (Fig. 2). Based on this design, the user can only continue on to the next section or take a break and continue later from the same place.

The program includes both an *assessment protocol* and a *treatment protocol,* which includes three therapeutic components (psychoeducation, exposure, and overlearning), following the guidelines for good clinical practice [1, 41]. First, *psychoeducation* includes specific information related to FP (i.e., how many people are affected, or how the problem begins and is maintained), using text, vignettes, and illustrations. Then, the *exposure* component is provided by the system, depending on the patient’s anxiety level recorded in the assessment (based on the FFQ-II questionnaire scores [61]). *Exposure* is performed through six scenarios composed of significant stimuli such as images and real sounds related to flying situations: (1) flight preparation, (2) airport, (3) boarding and taking off, (4) the central part of the flight, (5) the airplane’s descent, approach to the runway, and landing, (6) sequences with images and auditory stimuli related to plane crashes. The system advances to the next scenario when the user overcomes the current stage (anxiety level below 3 on a scale ranging from 0 “no anxiety” to 10 “high anxiety”). Thus, the program reacts in real time to each patient’s needs on the exposure task. After completing all the exposure scenarios, *overlearning* is offered as additional exposure, and participants may choose the scenarios that they want to confront based on their needs - from the same scenarios as in the exposure stage (except the air crash news scenario) - with a higher degree of difficulty, simulating storm conditions and turbulence.

All participants were advised to participate in about two exposure scenarios per week, taking a few days off between sessions. It was estimated that the treatment could be completed in three or four weeks, with a maximum period of six weeks. However, each participant was free to advance at his/her own pace. Furthermore, after the program, all the patients were encouraged to take a real flight. Although it was recommended that the flight be taken within two weeks after finishing the treatment, participants could schedule it based on their possibilities. The cost of the flight was paid for by each participant. *NO-FEAR Airlines* provides guidelines to cope with this test flight through downloadable material (pdf files). At the end of the treatment, the system provides post-treatment and follow-up assessments.

**Fig. 3:**
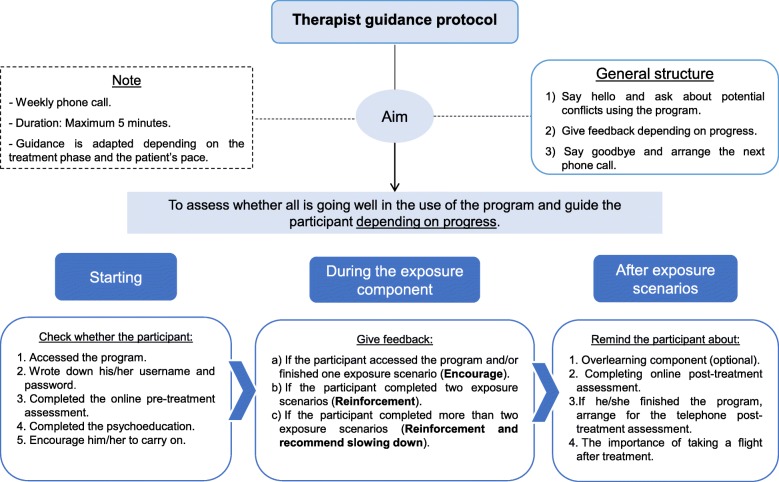
Therapist guidance protocol

The program described was implemented in two formats: 1) NO-FEAR Airlines totally self-applied. Participants received the completely self-applied treatment and only automatic support was provided throughout the program (i.e., automatic reinforcement after each exposure scenario). Technical assistance (i.e., web accessibility problems or forgotten password) was provided, if necessary. 2) NO-FEAR Airlines with therapist guidance. In this case, participants also self-administered the treatment via the Internet and received minimal telephone support from the therapist. Therapist guidance consisted of a brief weekly phone call (maximum 5 min), to assess and guide the participant’s progress by providing feedback and reinforcement until s/he had finished the treatment. Thus, patients could receive up to 6 telephone calls, and so they had a maximum of 30 min of therapeutic support. In addition, the therapist checked for any problems and reminded the participant about the recommended treatment pace. Guidance content was standardized; although it could be tailored depending on patients’ needs (see Fig. 3 for details). However, support calls could not include any additional clinical content. Telephone guidance was provided by trained and experienced psychologists.

### Outcomes

Assessments were conducted via phone call, a commercial online survey system (www.surveymonkey.com), and the *NO-FEAR Airlines* program. Participants were assessed at baseline, post-treatment, and 3- and 12-month follow-ups. A detailed description of the measures and sources of assessment can be found in the study protocol [55]. Measures included in this study were as follows:

*Diagnostic interview*. The Anxiety Disorders Interview Schedule for DSM-IV-TR (ADIS-IV) [62]. *Primary outcomes*. The Fear of Flying Questionnaire-II (FFQ-II) [61]; The Fear of Flying scale (*FFS*) [63]. *Secondary outcomes*. Fear and Avoidance Scales (adapted from Marks & Mathews [64]); The Clinician Severity Scale (adapted from Di Nardo, Brown & Barlow [65]); The Patient’s Improvement Scale (Adapted from the Clinical Global Impression Scale [66]). *Measures related to FP.* The duration of the problem; how many times the patient has taken a flight; whether safety behaviors were used (e.g., alcohol intake, distraction); and whether the participant has had any negative experiences with flying.

### Sample size

Power calculations and Internet attrition rates (30%) [67, 68] indicated that a sample size of a minimum of 57 participants (19 in each group) would be sufficient to detect a large effect size (*d* = 1) with a power of 0.80 and an alpha of 0.05, based on a similar study [22] and recent systematic reviews [32].

### Statistical methods

Group differences in participants’ socio-demographic and clinical data at baseline were examined using one-way analyses of variance (ANOVAs) for continuous data and chi-square tests (*χ*^2^) for categorical variables. Intent-to-treat (ITT) mixed-model analyses without any ad hoc imputations were conducted to handle missing data due to participant drop-out [69]. This approach uses all available data, it does not involve any substitution of missing values with supposed or estimated values, and it does not assume that the last measurement is stable (the last observation carried forward assumption) [70, 71]. Mixed-model analyses are appropriate for RCTs with multiple time points and pre-to post-only designs [56]. The assumption that data were missing completely at random (MCAR) was evaluated using Little’s MCAR test. A linear mixed-model for each outcome measure was implemented using the MIXED procedure with one random intercept per subject. An identity covariance structure was specified to model the covariance structure of the random intercept. For each outcome, *time* was treated as within-group factor and *group* as a between-group factor. Significant effects were followed up with pairwise comparisons (adjusted by Bonferroni correction). Separate mixed-model analyses were conducted to compare changes from baseline in each intervention, including both the 3- and 12-month follow-ups. Effect sizes (Cohen’s *d*) were calculated for within- and between-group comparisons [72–75]. The reliable change index (RCI) [76] for primary outcome measures (FFQ-II and FSS) was calculated based on the completer sample at post-treatment. Chi-square tests were performed to evaluate group differences in RCI rates, behavioral outcomes (post-treatment flights and safety behaviors), and participant diagnostic status for completers at post-treatment and two follow-ups. All statistical analyses were conducted using IBM SPSS Statistics for Windows, version 23.

## Results

### Participant flow and attrition

The recruitment started on September 2015 and ended on August 2016. Initially, as the flow diagram shows (Fig. 1), 146 people were interested in the study, and 85 of them were assessed for eligibility criteria. At this stage, 16 participants were excluded from the study. Finally, 69 participants were included in the study, and they were randomly allocated to each experimental condition (*NO-FEAR Airlines* totally self-applied, *n* = 23; *NO-FEAR Airlines* with therapist guidance, n = 23; and WL, n = 23). Of those who started the program (*n* = 46), 13 participants (28.26%) withdrew from the treatment conditions. No significant differences were found between the two treatment groups in attrition rates at post-treatment. At 3-month follow-up, a total of 22 participants (47.83%) completed the assessment, with significant differences between groups (χ^2^ (1) = 5.576; *p* = .018) (see Fig. 1 for details). Finally, at 12-month follow-up, a total of 20 participants completed the assessment (28.99%), but no significant differences between groups were obtained (χ^2^ (1) = 1.415; *p* = .234). The last participant completed the 12-month follow-up in December 2017. In the WL control group, data from 23 participants were obtained after they had spent 6 weeks on the waiting list (100% retention). Data were missing completely at random (MCAR) (*p* > .05).

### Baseline data and participant characteristics

Table 1 shows participants’ sociodemographic and clinical data for each group. No statistically significant differences were found on any sociodemographic or participant’s data, or on primary and secondary outcomes at baseline. Overall, participants came from Spain (91.3%, *n* = 63), Colombia (2.9%, *n* = 2), Chile (1.4%, *n* = 1), Cuba (1.4%, n = 1), USA (1.4%, n = 1), and Italy (1.4%, n = 1). They were not receiving stable medication, except four participants who were receiving anxiolytics to treat anxiety symptoms, with no significant differences between groups. There were no increases and/or changes in the medication intake during the study.

**Table 1:** Demographic and participant data

	*NFA* (*n* = 23)	*NFA + TG* (n = 23)	WL (n = 23)	Statistics
*Age*	36.30 (8.14) [range = 21–50]	38.87 (13.56) [range = 20–65]	34.13 (9.00) [range = 21–53]	F(2,69) = 1.173 *p* = .316
*Sex*				
*Male*	8 (34.8%)	6 (26.1%)	5 (21.7%)	χ^2^(2) = 1.02 *p* = .601
*Female*	15 (65.2%)	17 (73.9%)	18 (78.3%)	
*Marital status*				
*Married*	12 (52.2%)	11 (47.8%)	10 (43.5%)	χ^2^ (4) = .74 *p* = .946
*Single*	10 (43.5%)	11 (47.8%)	11 (47.8%)	
*Divorced/Separated*	1 (4.3%)	1 (4.3%)	2 (8.7%)	
*Educational status*				
*Primary studies*	–	–	1 (4.3%)	χ^2^(4) = 5.49 *p* = .240
*Secondary school*	2 (8.7%)	7 (30.4%)	5 (21.7%)	
*University education*	21 (91.3%)	16 (69.6%)	17 (73.9%)	
*Employment status*				
*Student*	4 (17.4%)	4 (17.4%)	1 (4.3%)	χ^2^(8) = 12.41 *p =* .134
*Unemployed*	4 (17.4%)	4 (17.4%)	1 (4.3%)	
*Employed*	15 (65.2%)	12 (52.2%)	2 (8.7%)	
*Retired*	–	3 (13%)	19 (82.6%)	
*Medication*				
*Yes*	1 (4.3%)	2 (8.7%)	1 (4.3%)	χ^2^(2) = .53 *p* = .767
*No*	22 (95.7%)	21 (91.3%)	22 (95.7%)	
*Experience Flying?*				
*Yes*	21 (91.3%)	20 (87%)	22 (95.6%)	χ^2^(2) = 1.20
*No*	2 (8.7%)	3 (13%)	1 (4.4%)	*p* = .547
*Duration of Phobia*				
*< 6 months*	1 (4.3%)	1 (4.4%)	1 (4.4%)	χ^2^(8) = 2.145
*6–12 months*	0 (0%)	1 (4.4%)	1 (4.4%)	*p* = .976
*1–5 years*	3 (13%)	3 (13%)	3 (13%)	
*6–10 years*	7 (30.4%)	7 (30.4%)	7 (30.4%)	
*> 11 years*	12 (52.2%)	11 (47.8%)	11 (47.8%)	
*Nationality*				
*Spanish*	21	20	22	χ^2^(2) = 1.09 *p* = .578
*Foreign*	2	3	1	

Means and standard deviations (SD) are represented for age (years). NFA. NO-FEAR Airlines totally self-applied without therapist guidance. NFA + TG. NO-FEAR Airlines with Therapist guidance. WL. Waiting list

### Effectiveness: Change in primary and secondary outcomes from pre- to post-treatment

#### Primary outcomes

The main effects of Time and Group were qualified by a significant interaction for *FFQ-II* (F(2, 57.48) = 21.151; *p* < .001) and *FFS* (F(2, 57.58) = 29.301; *p* < .001). For both primary outcomes, within-group comparisons indicated significant pre-to-post reductions in the two treatment groups with large effect sizes, and non-significant changes in the WL control group (see Table 2 for details). Between-group comparisons revealed that participants who received the treatment (with and without therapist guidance) scored lower at post-treatment, compared to the WL group, with large effect sizes (see Table 3). There were no statistically significant differences between the two treatment groups at post-treatment (all *p* > .05).

**Table 2:** Means, standard deviations and within-group effect sizes for primary and secondary outcomes at pre-, post-treatment, and 3-month follow-up

	*NO-FEAR Airlines* totally self-applied							*NO-FEAR Airlines* with therapist guidance							Waiting list		
	Pre (*n* = 23)	Post (*n* = 17)	3FW (*n* = 15)	12FW (*n* = 12)	Pre vs. Post	Pre vs. 3FW	Pre vs. 12FW	Pre (*n* = 23)	Post (*n* = 15)	3FW (*n* = 7)	12FM (n = 8)	Pre vs. Post	Pre vs. 3FW	Pre vs. 12FW	Pre (*n =* 23)	Post (*n* = 23)	Pre vs. Post
					*d* (95%CI)	*d* (95%CI)	*d* (95%CI)					*d* (95%CI)	*d* (95%CI)	*d* (95%CI)			*d* (95%CI)
FFQ-II	202.57 (33.54)	149.29 (46.35)	132.85 (46.35)	135.70 (57.74)	*d* = 1.53 (.94, 2.13)	*d* = 2.01 (1.29, 2.71)	*d* = 1.85 (.87, 2.83)	215.19 (29.69)	138.53 (55.53)	124.57 (67.85)	92.71 (63.81)	*d* = 2.49 (1.55, 3.43)	*d* = 2.95 (1.87, 4.02)	*d =* 3.67 (1.01, 6.33)	205.35 (49.64)	204.52 (48.54)	*d* = .02 (−.18, .30)
FFS	65.91 (6.93)	48.24 (9.80)	44.53 (11.49)	48.09 (12.07)	*d* = 2.46 (1.63, 3.29)	*d* = 2.98 (1.94, 4.02)	*d* = 2.39 (1.08, 3.71)	66.96 (6.94)	47.75 (17.00)	44.00 (14.93)	38.25 (13.70)	*d* = 2.67 (1.75, 3.59)	*d* = 2.88 (.79, 5.00)	*d* = 3.61 (1.09, 6.14)	63.96 (12.28)	65.18 (11.85)	*d =* −.10 (−.33, .13)
TB																	
Fear	9.26 (.86)	5.29 (1.36)	5.73 (2.02)	5.67 (1.92)	*d* = 4.46 (3.00, 5.91)	*d* = 3.91 (2.38, 5.43)	*d =* 3.88 (1.99, 5.77)	9.26 (.86)	5.13 (2.33)	4.29 (2.93)	3.78 (3.11)	*d* = 4.64 (3.12, 6.16)	*d* = 5.03 (1.30, 8.75)	*d* = 5.64 (1.93, 9.35)	9.22 (1.20)	8.17 (2.41)	*d* = .84 (.50, 1.19)
Avoidance	8.00 (2.43)	4.18 (2.33)	3.33 (2.82)	3.67 (3.00)	*d* = 1.52 (.92, 2.16)	*d* = 1.82 (.98, 2.65)	*d =* 1.66 (1.75, 2.56)	8.57 (1.62)	4.25 (3.55)	2.29 (2.98)	1.63 (2.93)	*d* = 2.57 (1.61, 3.54)	*d* = 3.37 (.57, 6.17)	*d =* 4.09 (1.32, 6.85)	8.04 (2.84)	8.14 (2.83)	*d* = −.03 (−.17, .10)
Belief	8.74 (1.01)	5.35 (2.15)	5.13 (3.25)	5.25 (2.38)	*d* = 3.24 (2.14, 4.35)	*d* = 3.38 (1.93, 4.83)	*d =* 3.21 (1.54, 4.89)	8.78 (1.51)	4.88 (2.66)	3.57 (2.88)	2.25 (2.71)	*d* = 2.49 (1.53, 3.45)	*d* = 3.00 (.51, 5.49)	*d =* 3.84 (4.20, 6.49)	8.83 (1.61)	8.77 (1.63)	*d* = .04 (−.17,.24)
Severity	7.30 (.56)	4.18 (1.51)	3.53 (1.77)	3.38 (1.69)	*d* = 5.38 (3.64, 7.11)	*d* = 6.37 (3.76, 8.97)	*d =* 6.51 (3.41, 9.62)	7.40 (.72)	4.19 (2.37)	2.86 (1.77)	2.17 (2.04)	*d* = 4.30 (2.85, 5.76)	*d* = 5.49 (1.43, 9.54)	*d =* 6.46 (2.27, 10.64)	7.26 (1.10)	7.27 (1.20)	*d* = −.01 (−.21, .20)
Improvement	–	5.41 (.80)	5.67 (.90)	5.42 (.90)	–	–	–	–	5.44 (1.09)	6.14 (.90)	6.13 (1.25)	–	–	–	–	3.91 (.68)	–

Means and standard deviations (SD) are represented for each primary and secondary outcome measures. Pre. Pre-treatment. Post. Post-treatment. 3FW. 3-month follow-up. 12FW. 12-month follow-up. FFQ-II. Fear of Flying questionnaire. FFS. Fear of Flying Scale. TB. Target Behavior. Belief. Degree of belief on the main irrational thought related to the target behavior. Severity. The Clinician Severity Scale. Improvement. The Patient’s Improvement Scale

**Table 3:** Between-group comparisons and effect sizes on primary and secondary outcome measures at post-treatment and 3- and 12-month follow-up

		Post-treatment		3-month follow-up		12-month follow up	
		Mean dif.	*d* (95%CI)	Mean dif.	*d* (95%CI)	Mean dif.	*d* (95%CI)
FFQ-II							
	NFA vs. WL	−57.78***	*d =* −1.13 (−1.81, −.46)	–		–	
	NFA + TG vs. WL	−66.45***	*d* = − 1.27 (− 1.98, −.56)	–		–	
	NFA vs. NFA + TG	8.67	*d* = .23 (−.46, .93)	−.25	*d* = .15 (−.77, 1.07)	36.90	.68 (−.24, 1.60)
FFS							
	NFA vs. WL	− 17.23***	*d* = − 1.51 (−.2.13, −.71)	–		–	
	NFA + TG vs. WL	−17.68***	*d* = − 1.21 (− 1.92, −.49)	–		–	
	NFA vs. NFA + TG	.46	*d* = .03 (−.66, .73)	−.53	*d* = .04 (−.86, .94)	9.11	.74 (−.18, 1.66)
TB							
*Fear*	NFA vs. WL	−.2.86***	*d* = − 1.40 (−2.10, −.69)	–		–	
	NFA + TG vs. WL	−3.06***	*d* = − 1.25 (− 1.96, −.54)	–		–	
	NFA vs. NFA + TG	.21	*d* = .08 (−.06, .78)	1.08	*d* = .34 (−.01, .68)	1.77*	.74 (−.16, 1.66)
*Avoidance*							
	NFA vs. WL	−3.83***	*d* = − 1.48 (− 2.19, −.76)	–		–	
	NFA + TG vs. WL	−3.83***	*d* = − 1.21 (− 1.91, −.51)	–		–	
	NFA vs. NFA + TG	.004	*d* = −.02 (−.71, .66)	.66	*d* = .35 (−.55, 1.25)	1.70	.66 (−.26, 1.57)
*Belief*							
	NFA vs. WL	−3.42***	*d* = − 1.79 (− 2.54, − 1.04)	–		–	
	NFA + TG vs. WL	−3.95***	*d* = − 1.80 (− 2.56, − 1.04)	–		–	
	NFA vs. NFA + TG	.53	*d* = .19 (−.49, .87)	1.08	*d* = .48 (−.43, 1.40)	2.90*	1.14 (.18, 2.11)
Severity							
	NFA vs. WL	−3.07***	*d* = − 2.25 (− 3.10, − 1.45)	–		–	
	NFA + TG vs. WL	−3.11***	*d* = − 1.69 (− 2.44, −.94)	–		–	
	NFA vs. NFA + TG	.04	*d* = −.01 (−.69, .68)	.42	*d* = .36 (−.54, 1.39)	1.173	.63 (−.28, 1.55)
Improvement							
	NFA vs. WL	1.50***	*d* = 2.00 (1.23, 2.46)	–		–	
	NFA + TG vs. WL	1.53***	*d* = 1.71 (.96, 2.46)	–		–	
	NFA vs. NFA + TG	−.26	*d* = −.03 (−.71, .65)	− 1.64	*d* = −.51 (− 1.42, .40)	−.58	−.65 (− 1.56, .27)

*NFA* NO-FEAR Airlines totally self-applied without therapist guidance *NFA + TG* NO-FEAR Airlines with Therapist guidance, *Mean dif* Mean differences, *WL* Waiting list, *d.* Cohen’s *d.* *CI* Confidence interval, *FFQ-II* Fear of Flying questionnaire, *FFS* Fear of Flying Scale, *TB* Target Behavior Belief. Degree of belief on the main irrational thought related to the target behavior. Severity. The Clinician Severity Scale. Improvement. The Patient’s Improvement Scale

* *p* < .05. *** *p* < .001

#### Secondary outcomes

Regarding secondary outcome measures, the main effects of Time and Group were qualified by a significant interaction for the *Clinician Severity Scale* (F(2, 62.13) = 34.867; *p* < .001) and the *Fear and Avoidance Scales* related to the main target behavior (taking a flight) [Fear (F(2, 64.54) = 17.906; *p* < .001), Avoidance (F(2, 57.52) = 21.242; *p* < .001), and the degree of Belief in the main catastrophic thought (F(2, 60.14) = 24.771; *p* < .001)]. Results of within-group comparisons showed significant reductions on these measures in the two treatment groups, corresponding to large effect sizes (see Table 2). There were no significant changes in the WL group, except for *Fear related to the target behavior* (*p* < .05; *d* = .84 [CI95% .50, 1.19]). At post-treatment, between-group comparisons revealed that the two treatment groups scored significantly lower on all the secondary outcome measures, compared to WL, and non-significant differences were found between the two ways of delivering the Internet-based treatment (with and without therapist guidance) (Table 3).

For the *Patient’s Improvement Scale *assessed at post-treatment, results showed a significant main effect of Group (F(2, 52) = 20.807; *p* < .001), indicating that the improvement achieved and reported by patients was statistically higher in the Internet-based treatment groups (with and without therapist guidance) compared to WL with large effect sizes (Table 3). The differences between the two treatment groups were not statistically significant (all *p* > .05).

### Maintenance of treatment gains at 3- and 12-month follow-ups

Separate linear mixed-model analyses yielded a significant main effect of Time on all the primary and secondary outcomes (all *p*s < .001), except for the *Patient’s improvement Scale*. Overall, within-group comparisons revealed significant changes from baseline to the 3- and 12-month follow-ups in the two treatment groups, indicating maintenance of the treatment gains. In addition, taken together, within-group effect sizes were higher for the pre-treatment to 3- and 12-month follow-up changes than for the pre- to post-treatment change (see Table 3). No significant interaction effect (Time by Group) was found on primary and secondary measures, except for the degree of *Belief in the main catastrophic thought* related to the main target behavior (taking a flight) (F(2, 87.03) = 2.868; *p* < .001), indicating that the Internet-based treatment with therapist guidance group scored lower than the completely self-applied group at 12-month follow-up (*d* = 1.14; 95% IC (.18, 2.11)). Separate between-group comparisons also revealed significant differences between the two Internet-based treatment groups (with and without therapist guidance) at 12-month follow-up on *Fear* *related to the main target behavior*, showing lower fear scores in the *NO-FEAR Airlines* with therapist guidance group (Table 3). No other significant between-group differences were found at 3- and 12-month follow-ups.

### Clinically meaningful improvement: Reliable change

**Fig. 4:**
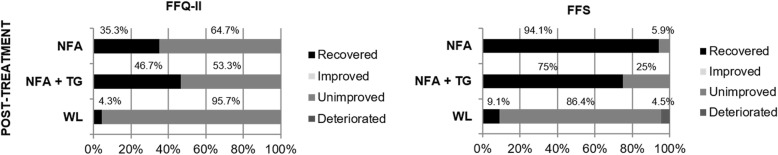
Reliable change. Percentage of the completer sample in each condition corresponding to recovered, improved, unimproved or deteriorated. FFQ-II. Fear of Flying Questionnaire. FFS. Fear of Flying Scale. NFA. NO-FEAR Airlines. NFA + TG. NO-FEAR Airlines with therapist guidance. WL. Waiting list

Figure 4 presents the proportion of completers in each condition who were recovered, improved, unimproved, or deteriorated at post-treatment. At post-treatment, statistically significant differences were found between the three conditions in these percentages on the *FFQ-II* (χ^2^(2) = 9.82; *p* < .01) and the *FSS* (χ^2^(4) = 31.972; *p* < .001). Overall, participants who had received the Internet-based interventions (with and without therapist guidance) showed higher recovered percentage compared to WL.

### Diagnostic status and behavioral outcomes

Results for diagnostic status and behavioral outcome measures (post-treatment flights and safety behaviors) are shown in Table 4. Analyses revealed statistically significant differences between groups at post-treatment for FP diagnostic status, safety behaviors, and number of safety behaviors. Specifically, both treatment groups (with and without therapist guidance) scored lower than the WL group: they had a lower percentage of FP diagnosis, the number of participants who reported using safety behaviors was lower, and they used fewer safety behaviors. No statistically significant differences were found at 3- and 12-month follow-ups on diagnostic status or the behavioral outcome measures.

**Table 4:** Diagnostic status and behavioral outcome measures at post-treatment and 3-and 12-month follow-up

		NFA			NFA + TG			WL	Statistics
		Post (*n* = 17)	3FW (*n* = 15)	12FW (*n* = 12)	Post (*n* = 15)	3FW (*n* = 7)	12FW (*n* = 8)	Post (*n* = 23)	
*FP Diagnosis*	*Yes*	11	6	6	9	3	1	23	Post: χ^2^(2) =10.709; *p* < .01
	*No*	6	9	6	6	4	7	0	3FW: χ^2^(1) = .175; *p* = .676
									12FW: χ^2^(1) = 2.967; *p* = .085
*Post-treatment flights*	*Yes*	5	12	10	3	3	6	3	Post: χ^2^(2) = 1.514; *p* = .469
	*No*	12	3	2	12	4	2	20	3FW: χ^2^(1) = 1.257; *p* = .262
									12FW: χ^2^(1) = 1.257; *p* = .648
*Number of flights taken*	*0*	12	3	2	12	3	0	20	Post: χ^2^(6) = 3.059; *p* = .801
	*2*	4	9	5	3	2	3	0	3FW: χ^2^(4) = 3.101; *p* = .541
	*4*	1	2	2	0	2	3	2	12FW: χ2 (4) = 3.718; *p* = .446
	*6*	0	1	3*	0	0	2*	1	
*Safety behaviors*	*Yes*	2	7	5	1	2	4	18	Post: χ^2^ (2) = 25.408; *p* < .01
	*No*	15	8	7	14	5	4	5	3FW: χ^2^ (1) = 1.174; *p* = .279
									12FW: χ^2^ (1) = .038; *p* = .845
*Number of safety behaviors*	*0*	15	7	7	14	5	4	1	Post: χ^2^ (14) = 41.357; *p* < .001
	*1–3*	2	6	4	1	1	3	16	3FW: χ^2^ (4) = 4.982; *p =* .289
	*4–6*	0	0	1	0	1	1	5	12FW: χ^2^ (4) = .950; *p* = .917
	*7–9*	0	0	0	0	0		1	

NFA. *NO-FEAR Airlines* totally self-applied**.** NFA + TG**.** *NO-FEAR Airlines* with therapist guidance. FP. Flying Phobia. Post. Post-treatment. 3FW. 3-month follow-up. 12FW. 12-month follow-up.

*from 6 to 12 flights taken

## Discussion

The aim of this study was to investigate the effectiveness of an Internet-based exposure treatment for FP (*NO-FEAR Airlines*) compared to a WL control group in an RCT. Overall, the data revealed that the self-applied online intervention (with and without therapist guidance) was effective in treating FP, compared to the WL, with large between-group effect sizes at post-treatment. Results showed a statistically significant change from pre to post treatment on all primary and secondary outcome measures, corresponding to large within-group effect sizes in both Internet-based treatment groups. Regarding the diagnostic status and reliable change indexes, significant improvements were found in the two treatment groups compared to the WL. In addition, these treatment gains were maintained at 3- and 12-month follow-ups, and overall effect sizes were larger than those obtained for the pre-to-post change. These findings are consistent with previous studies showing the efficacy of computer-assisted exposure programs for FP treatment [21, 22].

An important research issue addressed in this study involves the use of the Internet to deliver self-administered exposure to the feared stimuli. *NO-FEAR Airlines* includes self-administered exposure scenarios composed of images and real sounds to provide systematic exposure through the computer. Therefore, results from the present study show that the combination of new technologies and self-help procedures is a useful clinical tool for FP treatment, as authors have also found for fear of public speaking [43]. It is also worth highlighting that, in addition to being effective, *NO-FEAR Airlines* seems to be well accepted by participants because none of the participants refused to start the treatment when they were informed about the procedure. This fact is especially relevant because it might suggest that Internet-based exposure treatment is a useful alternative to in vivo exposure, providing a less frightening way for participants to confront their fears [44]. As stated above, to date, most of the Internet-based treatments that include the exposure technique provide guidelines to face the feared stimuli through downloadable pdf files rather than through multimedia exposure scenarios. Given these findings, along with the recent advances and growing technological developments, further research is needed to improve Internet-based treatments that include exposure among their treatment components.

A secondary aim of this study was to explore the impact of therapist guidance. Our results overall indicate that providing a weekly phone call from a therapist did not significantly affect treatment outcomes at post-treatment or 3- and 12-month follow-ups. These findings are congruent with studies suggesting that therapist involvement might be minimized for FP treatment using computer-assisted exposure programs [22], and they contradict other findings showing the superiority of guided interventions over unguided interventions [48]. In this regard, it is important to note some issues that could explain our results. First, *NO-FEAR Airlines* was designed with linear navigation to make the treatment easier and ensure that participants only continue on to the next section (or exposure scenario) when they are ready. Moreover, after participants had overcome each exposure scenario, automated reminders and reinforcements were provided through text displayed on the screen. As various authors suggested, if the self-applied program is well structured and designed, and automated support is provided throughout the intervention, the role of human guidance might be less important [49–51, 77–79]. Second, all the participants received an initial phone call from a therapist who explained the research and conducted the screening procedures and telephone interviews. Research has highlighted that providing brief initial human contact before starting the treatment might be sufficient to produce an effect on the treatment outcomes, reducing the need for or impact of guidance throughout the treatment [80]. Third, therapist guidance might have different implications depending on the disorder addressed. Thus, it is also necessary to consider studies indicating that, whereas self-help interventions without therapist contact can be useful to treat simple psychological disorders (i.e., specific phobias), they may be insufficient for more severe mental disorders [43, 81]. Although there is recent evidence showing the utility of self-guided Internet Interventions for severe disorders (i.e., depression) [50], this issue remains unclear. Moreover, and despite our findings, research focusing on specific phobia treatment is scarce. There are only two RCT on Internet-based treatments but both involve direct self-exposure guided by the therapist from a distance, without addressing this topic [33, 34]. Fourth, therapist guidance may have significant effects in the long term instead of the short term, and therefore longer assessments are needed to reveal its effects. In our study, although generally there was no significant effect of therapist guidance on outcomes at post-treatment or 3- and 12-month follow-ups, a significantly lower belief in the main catastrophic thought and lower fear related to the main target behavior (taking a flight) were found in the supported group (NO-FEAR Airlines with therapist guidance) at 12-month follow-up. This result indicates that providing weekly therapist guidance through a brief phone call (i.e., 5 min) throughout the online intervention may help to reduce both the fear level and the degree of belief in catastrophic thoughts in the long term. However, more research is needed to continue to explore the impact of guidance, including long-term assessments. Furthermore, the therapist guidance that consisted of a brief weekly phone call of 5 min as maximum to provide feedback and reinforcement may be too short in order to achieve significant differences between the two interventions groups included in the present study (with or without therapist guidance). As far as we know, few studies have formally addressed the impact of dose–response-relationship regarding guidance on Internet interventions. As Baumeister et al. [46] pointed out in their systematic review study, only one study compared higher dose of guidance versus lower dose of guidance finding no statistically significant differences on symptoms severity at post-treatment. However, this study focused on the dose of guidance provided by email (one or three emails per week) and not on the guidance duration (i.e., minutes of the phone call).

In summary, our results point out the efficacy of *NO-FEAR Airlines* with and without therapist guidance. However, there are some limitations that should be mentioned. First, assessments were conducted online and via phone calls. Although several studies have shown the usefulness of Internet and telephone administered assessments [82–84], some authors suggest that psychometric properties may change [85]. Second, missing data at two follow-up assessments (3 and 12 months) were higher than expected (> 30%). Even though *NO-FEAR Airlines* sent automated reminders to participants and researchers to complete the assessment, we were unable to contact many of them, and they did not complete the 3- and 12-month follow-ups, limiting our conclusions about long-term treatment gains. Third, the interpretation of the behavioral outcome measures was compromised due to the low number of participants taking a flight after the treatment and the missing data rates, mentioned above. Given the importance of taking a flight after the treatment, outlined in several studies [8, 14, 86], further efforts are needed in this regard. We suggest that the use of persuasive technologies [78] to provide guidance and reinforcement after the treatment, as well as the use of short intervention packages (via web or mobile) to review or practice before taking a flight, could be useful in this endeavor. Despite that ADIS-IV was used as diagnosis interview and exclusion criteria were assessed in the screening conducted by telephone and throughout the protocol assessment included in *NO-FEAR Airlines*, no other diagnostic interview was systematically used to formally assess the presence of other mental disorder different from anxiety disorders. Finally, the study design and sample size calculations were mainly conducted as a superiority trial rather than as an equivalence trial [87–89]. Therefore, we can state that both ways of delivering the treatment (with and without therapist guidance) were effective for the treatment of FP, compared to the WL, but we cannot conclude that both conditions were equally efficacious. Future studies should be carried out to formally assess these issues.

As far as we know, this is the first RCT to investigate the effectiveness of an Internet-based intervention for FP and explore two ways of delivering the treatment (with and without therapist guidance). Overall, our findings indicate that FP can be effectively treated via the Internet. This study contributes to the literature on Internet-based interventions and adds additional data to the research on the use of computer-assisted exposure programs for FP treatment.

## Conclusions

The Internet-based treatment (*NO-FEAR Airlines*) was effective for treating FP, compared to a WL, regardless of whether therapist guidance was provided or not. *NO-FEAR Airlines* includes significant self-administered exposure scenarios composed of images and real sounds to enhance the exposure technique for FP treatment. This program helps to improve access to evidence-based psychological treatment and reach more people who may need it.

## Additional file

Additional file 1: NO-FEAR Airlines. Video about *NO-FEAR Airlines*, an Internet-based Exposure treatment for Flying Phobia. The video includes a description about the online program, how it works and on how it has been assessed in an RCT. (MP4 58734 kb)

## Abbreviations

ANOVA: Analysis of variance
APA: American Psychological Association
CGI: Clinical Global Impression scale
CI: Confidence Interval
CONSORT: Consolidated Standards of Reporting Trials
DSM-5: Diagnostic and Statistical manual for Mental Health Disorders-Version 5
FFQ-II: Fear of Flying Questionnaire-II
FFS: Fear of Flying scale
FP: Flying phobia
FW: Follow-up
ICTs: Information and Communication Technologies
ITT: Intention-to-treat
MI: Multiple Imputations
NFA: NO-FEAR Airlines
NICE: National Institute for Health and Clinical Excellence
RCT: Randomized Control Trial
TB: Target Behavior
TG: Therapist guidance
VRET: virtual reality exposure therapy
WL: Waiting list
